# Association between CILP and IL-1α polymorphisms and phenotype-dependent intervertebral disc degeneration susceptibility: A meta-analysis

**DOI:** 10.3389/fgene.2022.1005393

**Published:** 2022-10-06

**Authors:** Jiachen Liu, Yunxia Chen, Xiuqi Shan, Huan Wang

**Affiliations:** ^1^ Department of Orthopedics, Shengjing Hospital of China Medical University, Shenyang, China; ^2^ Department of Endocrinology, Cangzhou People’s Hospital, Cangzhou, China

**Keywords:** intervertebral disc degeneration, CILP, IL-1α, single nucleotide polymorphism, phenotype

## Abstract

**Background:** The relationship between CILP (1184T>C) and IL-1α(+889C/T) polymorphisms and intervertebral disc degeneration (IDD) have been explored in several studies but the results were conflicting. The aim of the study was to evaluate and synthesize the currently available data on the association between CILP (1184T>C) and IL-1α(+889C/T) polymorphisms and susceptibility of phenotype-dependent radiologic IDD (RIDD) and symptomatic intervertebral disk herniation (SIDH).

**Methods:** A computerized literature search was in PubMed, Cochrane Library, Embase, China National Knowledge Infrastructure database, and Web of Science. The pooled results were presented as odds ratios (ORs) with 95% confidence intervals (CIs). Moreover, the false-positive report probability (FPRP) test and trial sequential analysis (TSA) were applied to estimate the significant results.

**Results:** Our evidence demonstrated that IL-1α(+889C/T) was significant associated with RIDD (allele model: OR = 1.34, 95%CI 1.03–1.74, *p* = 0.029) and SIDH (allele model: OR = 1.28, 95% CI 1.03–1.60, *p* = 0.028). However, the results were not noteworthy under the FPRP test and TSA analysis. Additionally, CILP (1184T>C) polymorphism was significantly associated with RIDD with adequate evidence (allele model: OR = 1.27, 95% CI 1.09–1.48, *p* = 0.002) instead of SIDH.

**Conclusion:** The current meta-analysis illustrated firm evidence that CILP (1184T>C) polymorphism was significantly associated with the susceptibility of RIDD. However, the significant associations between IL-1α(+889C/T) and RIDD and SIDH were less credible. Thus, more multi-center studies with diverse populations were required to verify the results.

## Introduction

Low back pain (LBP), one of the most common muscle-skeletal diseases, contributes to lower life quality and even disability. Over 80% of elderly people are afflicted with LBP to some extent during their lifetime (Andersson, 1999). Intervertebral disc degeneration (IDD) is regarded as a major cause of LBP which has an extremely complex pathogenesis (Chou et al., 2011). Multiple risk factors such as environmental, diet, and genetic factors were reported to accelerate the pathogenesis process of IDD (Battié and Videman, 2006).

As a chondroid structure, the nucleus pulposus (NP) contains a luxuriant extracellular matrix (ECM) such as proteoglycans and collagen. Accumulating evidence has highlighted the important role of ECM homeostasis play in the development of IDD. The imbalance of ECM homeostasis may cause diverse changes in disc including height reduction, dehydration, endplate alteration, etc. (Roberts et al., 2006). Overexpression of proinflammatory and catabolic phenotypes is the major reason for the imbalance of ECM homeostasis in the NP (Sivan et al., 2014; Molinos et al., 2015). Overexpression of IL-1 in the degenerative disk tissues inhibited extracellular matrix synthesis and improved extracellular matrix degradation (Phillips et al., 2013; Li et al., 2017). Hypersensitivity to IL-1α in disc cells is a significant motivator for degeneration (Maeda and Kokubun, 2000). Cartilage intermediate layer protein (CILP) is sufficiently distributed in intervertebral discs and has a vital function in regulating the metabolism of ECM. CILP inhibits the production of cartilage matrix protein *via* the TGF-β1signaling pathway (Seki et al., 2005).

Given the vital function of the IL-1α and CILP in ECM metabolism, numerous candidate polymorphism loci correlated to IDD predisposition were identified. Various studies indicated that CILP (1184T>C) and IL-1α(+889C/T) polymorphisms could be significantly correlated with the susceptibility of IDD (Solovieva et al., 2004; Seki et al., 2005). However, recent studies failed to detect the association and even obtain the opposite conclusion (Virtanen et al., 2007; Kelempisioti et al., 2011; Abdollahzade et al., 2018; S M et al., 2018). More importantly, the results of previous systematic reviews are unreliable due to overlooking the phenotypes of IDD (Wang W. et al., 2016; Wang Z. et al., 2016; Rajasekaran et al., 2016). Under the circumstances, we aim to provide more exhaustive evidence and rigorous criteria to explore the correlation of IL-1α and CILP polymorphisms with radiologic IDD (RIDD) as well as symptomatic intervertebral disk herniation (SIDH).

## Material and methods

### Search strategy for literature

We performed a literature retrieval of PubMed, China National Knowledge Infrastructure database, Web of Science, Cochrane Library database, and Embase by using the following terms (“LDD” or “IDD” or “lumbar disease” or “lumbar degeneration” or “Intervertebral disc degeneration” or “herniation”) AND (“polymorphism” or “allele” or “variant”) AND (“IL-1α” or “CILP” or “IL-1A”). The bibliography of associated studies was also examined for additional correlative studies.

### Selection criteria

Studies included in the meta-analysis should be based on the following criteria: 1) case-control study; 2) the consequence was the investigation of the correlation between CILP (1184T>C) IL-1α(+889C/T) or rs1800587 or rs2073711and IDD or SIDH; 3) genotype frequencies were available to calculate an odds ratio (OR) with 95% confidence intervals (CIs); 4) grade criteria or phenotypes of IDD were described in detail.

The exclusion standards were listed as follows: 1) other types of study design; 2) genotype frequencies were not present in the articles; 3) studies contained overlapping data from previous research; 4) publication language was not English.

### Methodological quality assessment

The quality evaluation of each study was performed independently by two investigators (JCL and XQS) following the Newcastle-Ottawa Scale (NOS). Dissension between reviewers would be discussed to reach a consensus. Based on the NOS standard, only studies with calculating scores >5 were deemed of high methodological quality.

### Data extraction

Two reviewers independently extracted the following information on basis of a standard form: 1) Name of the First author 2) Publication information 3) Country 4) Ethnicity 5) Genotype frequencies 6) Numbers of cases and controls 7) Phenotypes of IDD.

### Statistical analysis

Stata 14.0 was applied to conduct the statistical analysis. The correlation strength was assessed by ORs and corresponding 95%Cl in five genetic models, including allele, domain, recessive, heterozygote, and homozygote models. THE Cochrane Q test and the I2 statistic test were performed in each model to assess the heterogeneity between studies, and significant heterogeneity was considered when I^2^ >50% or *p* < 0.05 (Higgins and Thompson, 2002). When significant heterogeneity existed, the pooled ORs would be estimated in the random-effects model; otherwise, the fixed-effects model (Mantel-Haenszel) was selected. Hardy-Weinberg equilibrium (HWE) was utilized to explore the genotype distribution of the population included in this research. Significant disequilibrium was defined by *p* < 0.05 in the chi-square test. Publication bias was quantitatively assessed through the Begg’s and Egger’s test and *p* < 0.05 represented a significant public bias. To evaluate the robustness of overall findings, sensitivity analysis was performed. Subgroup analysis was performed in RIDD to evaluate the genetic effect of IL-1α(+889C/T) and CILP (1184T>C)in different phenotypes of RIDD meanwhile stratified analysis was conducted in SIDH to test the genetic effect of CILP (1184T>C) in different ethnicity. False-positive report probability (FPRP) tests were performed to correct all significant pooled ORs. According to the calculating Excel spreadsheet (Wacholder et al., 2004), the values of FPRP were estimated at the prior probability of 0.01 to detect ORs of 1.5. FPRP >0.2 was thought to be a false significant result.

### Trail sequential analysis

In order to control type I and type II errors and predict the required information size (RIS) in the research, the trial sequential analysis (TSA) was carried out in the TSA software (version 0.9; www.ctu.dk/tsa) with a 5% type I error and 20% type II error (Roshanov et al., 2017). The trial sequential monitoring boundaries were based on the O'Brien-Fleming-spending function. If the cumulative Z-curve exceeds the RIS or TSA line, it suggested the significant association is credible. Otherwise, further studies are required to confirm the result (Wetterslev et al., 2017). If the cumulative Z-curve passes the futility boundary, the association is a true negative result.

## Results

### Study characteristics

**FIGURE 1:**
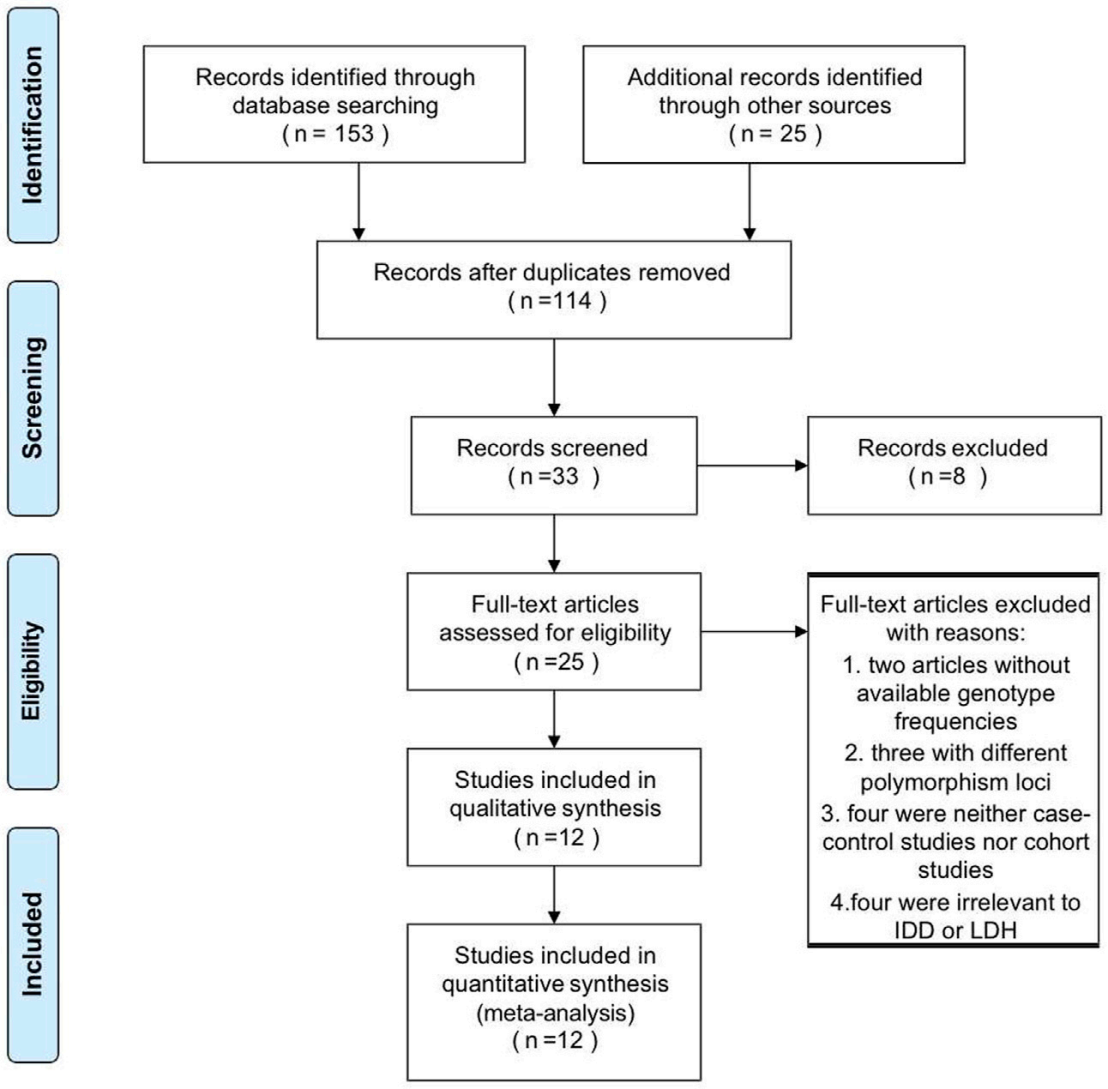
Flow diagram of the study identification and selection process.

The detailed literature search process was present in Figure 1. After duplicate removal and records screening, 25 articles were subjected to full-text screening. Conforming to the selection criteria, 13 studies were excluded with 12 literature remaining. Among them, six recruited studies evaluated the relationship between IL-1α rs1800587 and the risk of RIDD and SIDH in Caucasian, Mexica, and Chinese populations. Six studies consisting of Japanese, Chinese, Finland, and Indian populations were included in the analysis for CILP rs2073711. The characteristics and genotype frequencies of each study were shown in Table 1 meanwhile quality assessment was listed in Supplementary Table S1.

**TABLE 1:** Characteristic and genotype distributions of studies for the relationship between CILP and IL-1α polymorphisms and IDD.

Study	Year	Country	Ethnicity		Cases			Controls			HWE	Disease (Phenotype)
IL-1α(+889C/T)				Minor allele	CC	CT	TT	CC	CT	TT		
Solovieva(a) et al.	2004	Finland	Caucasian	T	11	21	6	34	51	8	0.059	RIDD (Solovieva grade)
Solovieva(b) et al.	2004	Finland	Caucasian	T	30	54	13	15	18	1	0.059	RIDD (Solovieva grade)
Karppinen et al.	2009	Finland	Caucasian	T	12	26	7	30	28	5	0.384	RIDD (Modic change)
Aparicio et al.	2011	Spain	Caucasian	T	22	25	3	63	61	5	0.016	SIDH
Serrano et al.	2014	Mexico	Mexican	T	51	45	4	55	35	10	0.835	RIDD (Modic change)
Chen et al.	2018	China	Han	T	87	78	28	102	81	14	0.327	SIDH
Abdollahzade et al.	2018	Iran	Caucasian	T	33	33	10	62	62	12	0.806	SIDH
CILP(1184T>C)												
Kelempisioti et al	2011	Finland	Caucasian	T	70	158	64	78	122	46	0.284	RIDD (Pfirrmann grade)
Seki et al	2005	Japan	Asian	C	34	162	271	18	185	451	0.217	SIDH
Virtanen(a) et al	2007	Finland	Caucasian	T	82	116	33	73	141	43	0.071	SIDH
Virtanen(b) et al	2007	China	Asian	C	4	88	249	4	83	251	0.115	RIDD (Schneiderman grade)
Min et al	2009	Japan	Asian	C	4	20	24	2	8	31	0.359	RIDD (Pfirrmann grade)
Min et al	2010	Japan	Asian	C	17	71	128	16	112	257	0.073	RIDD (Pfirrmann grade)
Bhat et al	2018	India	Caucasian	C	15	103	82	32	93	75	0.228	SIDH

a, b denote an independent study in the same article, respectively.

### IL-1α(+889C/T) polymorphism and RIDD susceptibility

**FIGURE 2:**
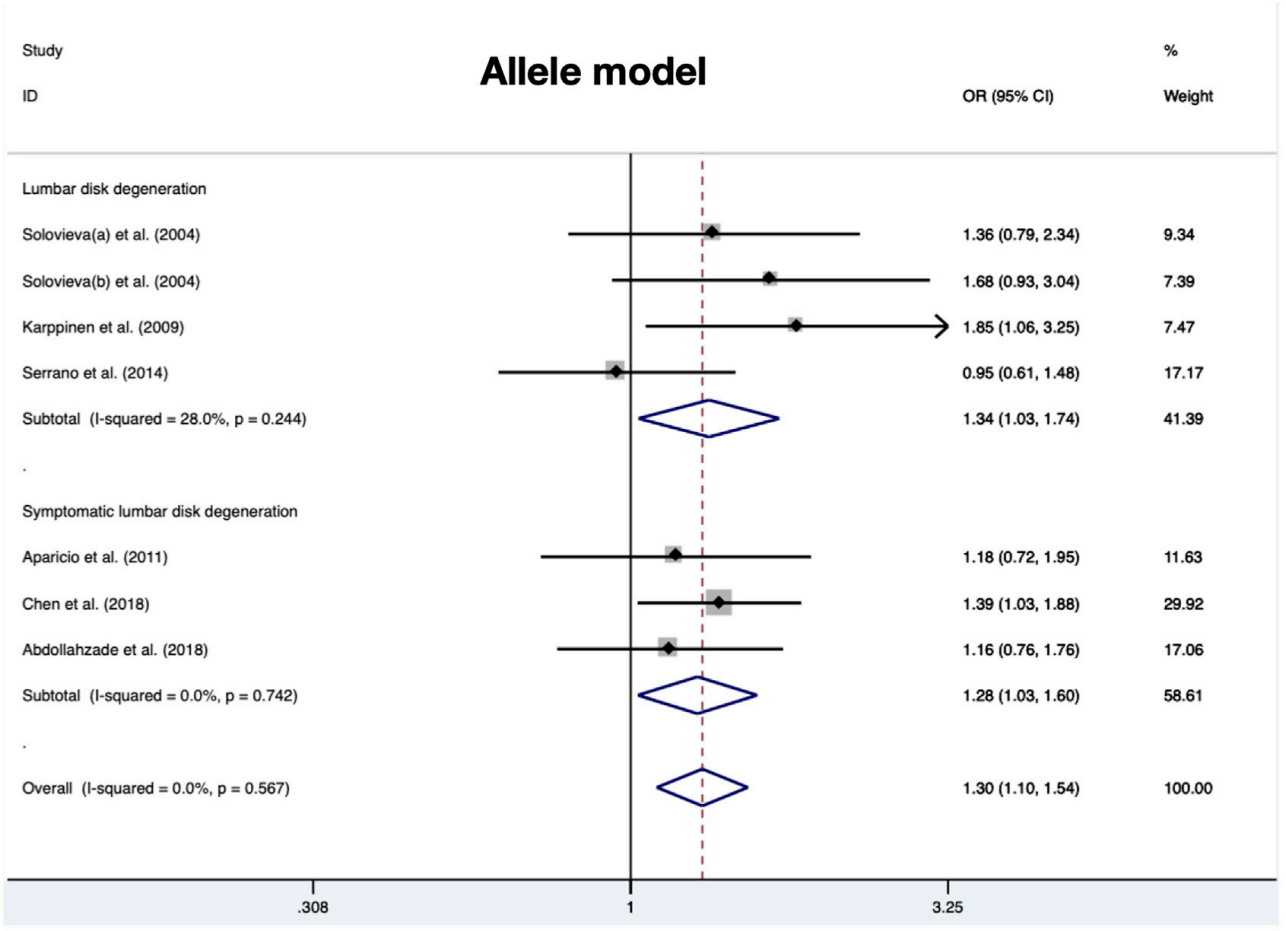
Meta-analysis for the relationship between IL-1α(+889C/T) polymorphism and the susceptibility of RIDD and SIDH (T vs. C).

**FIGURE 3:**
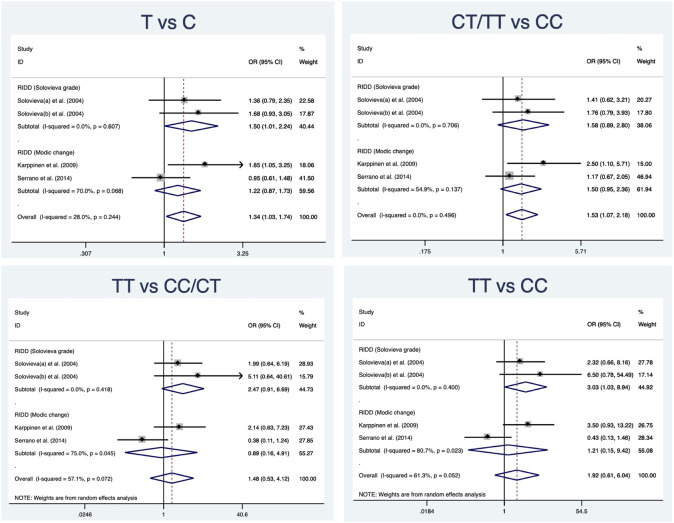
Subgroup analysis for the relationship between IL-1α(+889C/T) polymorphism and the susceptibility of RIDD.

A total of 280 cases and 290 controls were included to detect the association between IL-1α(+889C/T) polymorphism and RIDD susceptibility. Obvious heterogeneity was detected in the recessive and heterozygous models. The pooled OR confirmed significant associations in three models (allele model: OR = 1.34, 95%CI 1.03–1.74, *p* = 0.029, Figure 2; dominant model: OR = 1.53, 95%CI 1.07–2.18, *p* = 0.02; homozygous model: OR = 1.53, 95%CI 1.06–2.22, *p* = 0.025). No statistical significance was identified regarding the recessive and heterozygous models (Table 2). However, the FPRP test indicated the evidence was not noteworthy (FPRP >0.2). In subgroup analysis, it was shown that IL-1α(+889C/T) polymorphism was significantly correlated to RIDD (Solovieva grade) in the allele model but irrelevant to RIDD (Modic change) under any model (Figure 3).

**TABLE 2:** Meta-analysis of the association between genetic polymorphism and radiologic lumbar disk degeneration.

Genetic model	Overall effect		Heterogeneity		Publication bias		Analysis model	FPRP		
IL-1α(+889C/T)	OR (95%CL)	P	I^2^	P	Begg’s	Egger’s		0.1	0.01	0.001
T vs. C	1.34 (1.03–1.74)	0.029	28%	0.244	0.23	0.62	Fix	0.240	0.776	0.972
CT/TT vs. CC	1.53 (1.07–2.18)	0.02	0	0.496	0.133	0.189	Fix	0.268	0.801	0.976
TT vs. CC/CT	1.48 (0.53–4.12)	0.453	57.1%	0.072	0.764	0.894	Random			
TT vs. CC	1.92 (0.61–6.04)	0.266	61.3%	0.052	0.368	0.861	Random			
CT vs. CC	1.53 (1.06–2.22)	0.025	0	0.752	0.133	0.136	Fix	0.330	0.844	0.982
CILP (1184T>C)										
C vs. T	1.27 (1.09–1.48)	0.002	34.8%	0.203	0.881	0.825	Fix	0.02	0.182	0.691
CT/CC vs. TT	1.34 (1.10–1.63)	0.004	42.9%	0.154	0.764	0.887	Fix	0.034	0.280	0.797
CC vs. TT/CT	1.37 (0.97–1.93)	0.073	0	0.660	0.881	0.729	Fix			
CC vs. TT	1.68 (1.15–2.46)	0.007	0	0.737	0.652	0.763	Fix	0.198	0.730	0.965
CT vs. TT	1.29 (1.05–1.59)	0.015	37.7%	0.186	0.707	0.811	Fix	0.142	0.646	0.949

### IL-1α(+889C/T) polymorphism and SIDH susceptibility

Amount to 319 cases and 462 controls were integrated to investigate the risk score of IL-1α polymorphism for SIDH susceptibility. No significant heterogeneity was detected in any model. There was significant association detected in three models (allele model: OR = 1.28, 95%CI 1.03–1.60, *p* = 0.028, Figure 2; recessive model: OR = 1.92, 95%CI 1.16–3.17, *p* = 0.01; homozygous model: OR = 2.00, 95%CI 1.19–3.38, *p* = 0.009) (Table 3).

**TABLE 3:** Meta-analysis of the association between genetic polymorphism and symptomatic lumbar disk herniation.

Genetic model	Overall effect		Heterogeneity		Publication bias		Analysis model	FPRP		
IL-1α(+889C/T)	OR (95%CL)	P	I^2^	P	Begg’s	Egger’s		0.1	0.01	0.001
T vs. C	1.28 (1.03–1.6)	0.028	0	0.742	0.23	0.62	Fix	0.228	0.765	0.970
CT/TT vs. CC	1.23 (0.92–1.64)	0.167	0	0.876	0.133	0.189	Fix			
TT vs. CC/CT	1.92 (1.16–3.17)	0.01	0	0.801	0.764	0.894	Fix	0.367	0.864	0.985
TT vs. CC	2.00 (1.19–3.38)	0.009	0	0.780	0.368	0.861	Fix	0.380	0.871	0.986
CT vs. CC	1.10 (0.81–1.49)	0.535	0	0.929	0.133	0.136	Fix			
CILP (1184T>C)										
C vs. T	1.01 (0.62–1.66)	0.959	91.5%	0.00	0.881	0.825	Random			
CT/CC vs. TT	1.02 (0.60–1.73)	0.949	86.4%	0.001	0.764	0.887	Random			
CC vs. TT/CT	1.00 (0.36–2.76)	0.997	89.4%	0.000	0.881	0.729	Random			
CC vs. TT	0.98 (0.31–3.14)	0.972	90.9%	0.000	0.652	0.763	Random			
CT vs. TT	1.05 (0.69–1.60)	0.825	76.3%	0.015	0.707	0.811	Random			

### CILP (1184T>C) polymorphism and RIDD susceptibility

**FIGURE 4:**
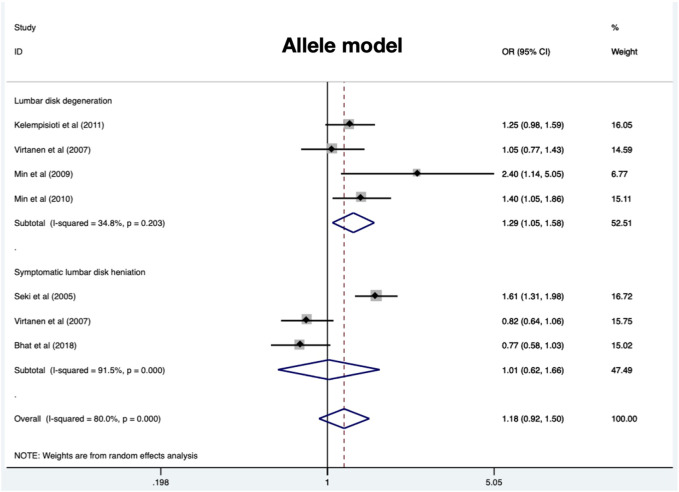
Meta-analysis for the relationship between CILP (1184T>C) polymorphism and the susceptibility of RIDD and SIDH (C vs. T).

**FIGURE 5:**
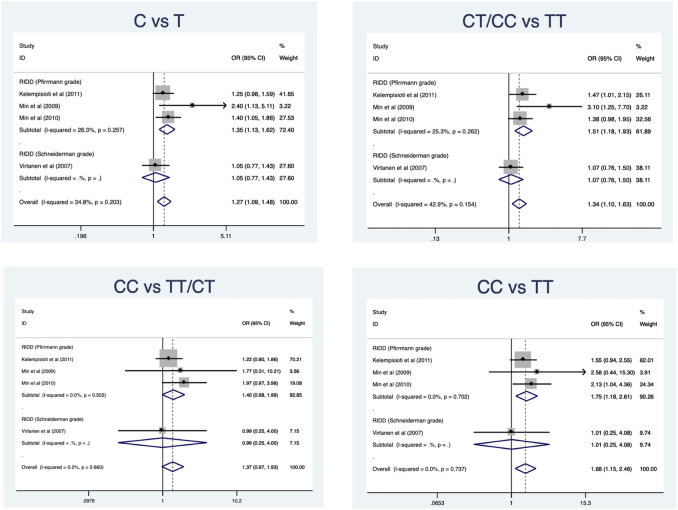
Subgroup analysis for the relationship between CILP (1184T>C) polymorphism and the susceptibility of RIDD.

Four eligible studies comprising 897 cases and 1010 controls (Virtanen et al., 2007; Min et al., 2009; Min et al., 2010; Kelempisioti et al., 2011) are included and overall results revealed a significant association in allele model (OR = 1.27, 95%CI 1.09–1.48, *p* = 0.002), dominant model (OR = 1.34, 95%CI 1.10–1.63, *p* = 0.004), and homozygous model (OR = 1.29, 95%CI 1.05–1.59, *p* = 0.015) (Table 2) (Figure 4). No significant heterogeneity was identified in all models. Subgroup analysis stratified by radiologic grade revealed significant associations between CILP (1184T>C) and RIDD (Pfirrmann grade) susceptibility in three models (allele model: OR = 1.35, 95%CI 1.13–1.62, *p* = 0.001; recessive model: OR = 1.51, 95%CI 1.18–1.93, *p* = 0.001; homozygous model: OR = 1.75, 95%CI 1.18–2.61, *p* = 0.006) but no association with RIDD (Schneiderman grade) susceptibility (Figure 5).

### CILP (1184T>C) polymorphism and SIDH susceptibility

A total of 898 cases and 1111 controls (Seki et al., 2005; Virtanen et al., 2007; S M et al., 2018) were recruited and the pooled results demonstrated no significant relationship in any models (Table 3) (Figure 4). All models suggested high heterogeneity. Subgroup analysis revealed a significant association between CILP (1184T>C) polymorphism and SIDH risk in the Asian population.

### Publication bias and sensitivity analysis

**FIGURE 6:**
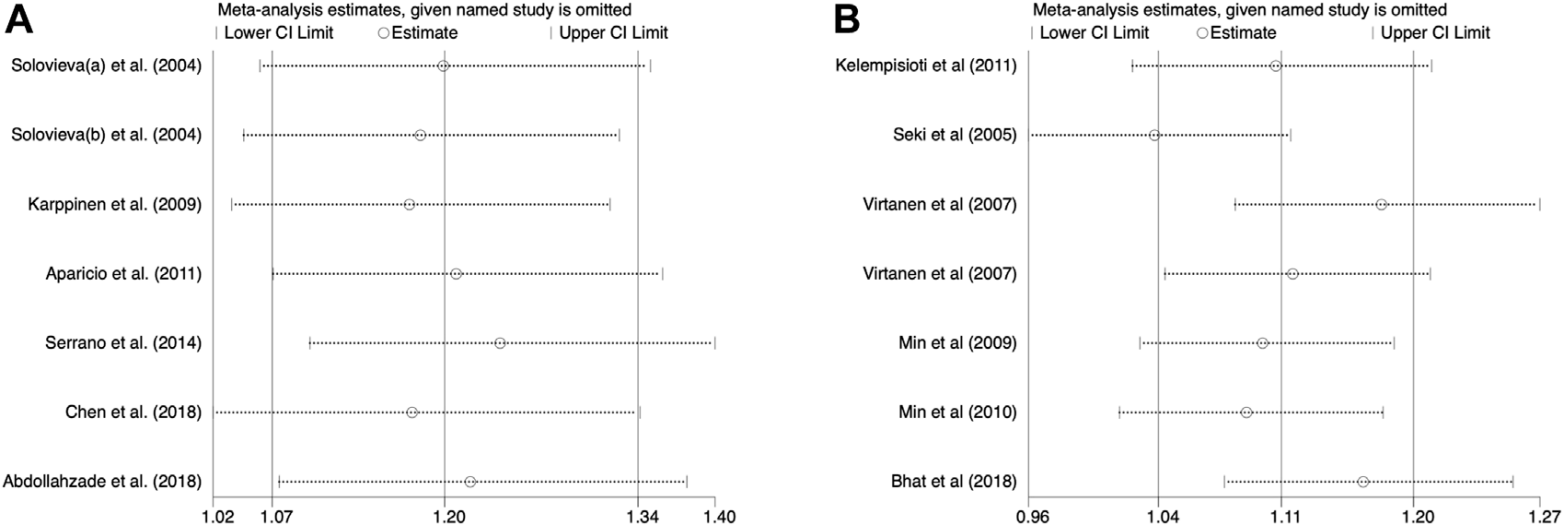
Sensitivity analysis. **(A)** Allele model for IL-1α(+889C/T) polymorphism. **(B)** Allele model for CILP (1184T>C) polymorphism.

A ruling one out in turn strategy was adopted to estimate the effect of exclusion for each study on pooling ORs. The overall estimates did not alter under any genetic model when each individual study was omitted in turn, demonstrating the robustness of the findings (Figure 6). Begg’s and Egger’s tests indicated the results were free from significant publication bias under any genetic model (Tables 2, 3).

### Significance test and TSA results

**FIGURE 7:**
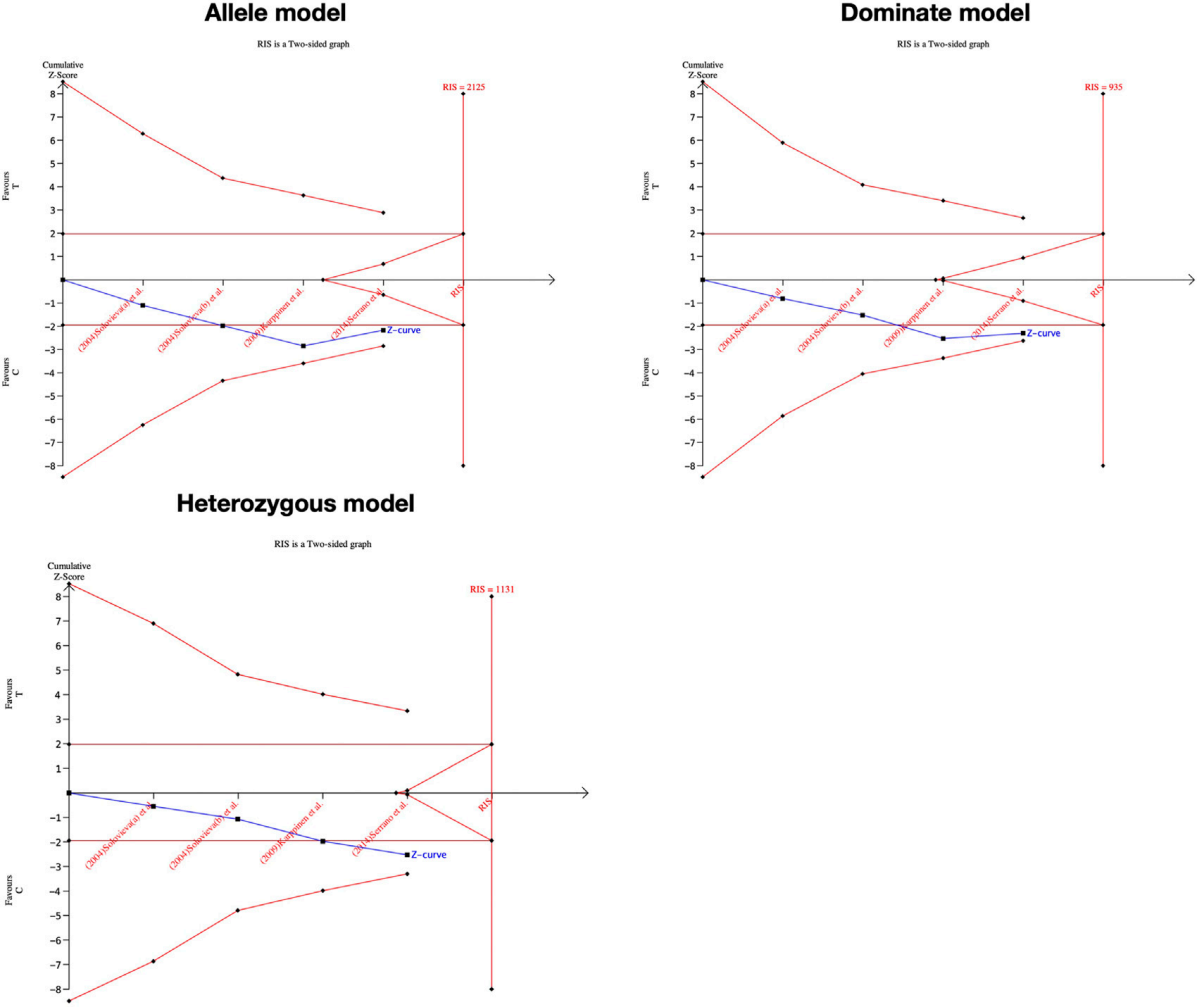
TSA for IL-1α(+889C/T) polymorphism and the susceptibility of RIDD.

**FIGURE 8:**
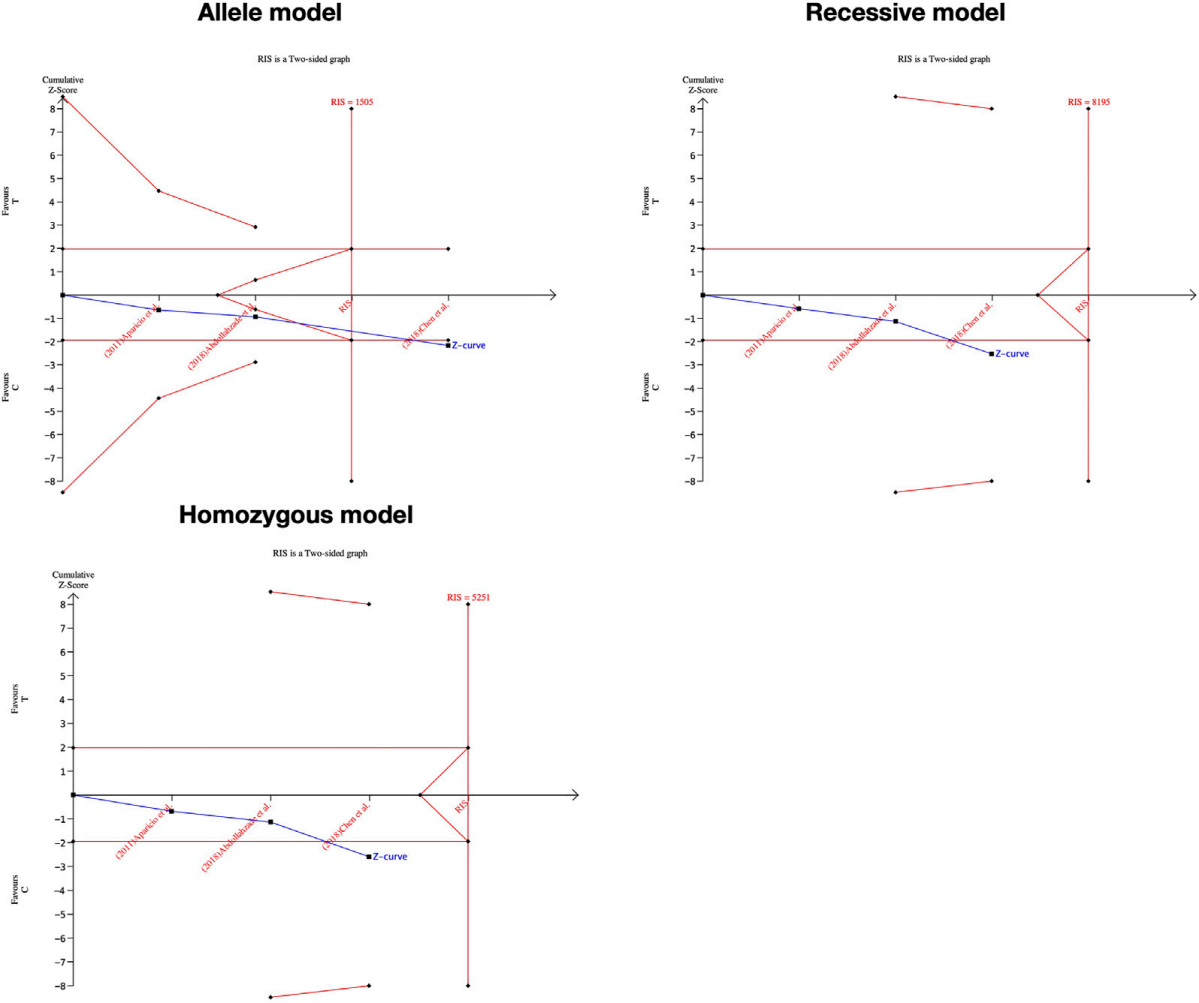
TSA for IL-1α(+889C/T) polymorphism and the susceptibility of SIDH.

**FIGURE 9:**
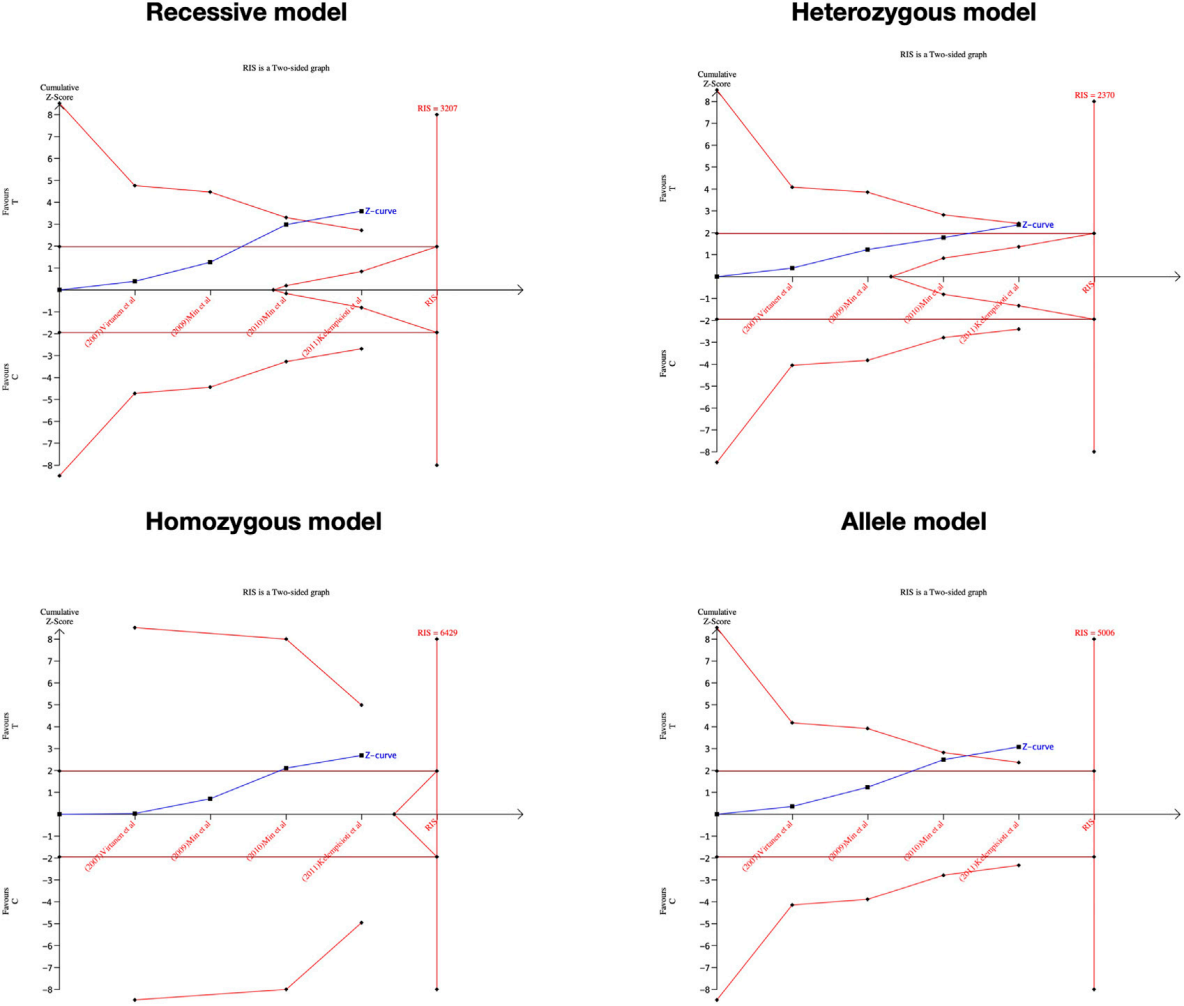
TSA for CILP (1184T>C) polymorphism and the susceptibility of RIDD.

We performed the FPRP test to investigate the false-positive results. The relationship between IL-1α(+889C/T) polymorphism and IDD and SIDH was not noteworthy (FPRP >0.2) in any model while the significant association between CILP (1184T>C) polymorphism and RIDD was considered a true positive result. The cumulative z-curve did not pass the TSA and RIS line for the association between IL-1α(+889C/T) polymorphism and IDD, indicating more studies are required (Figure 7). For IL-1α(+889C/T) polymorphism and SIDH, the cumulative z-curve crossed the futility line in the allele model and did not exceed the TSA and RIS boundary in the recessive and heterozygous model (Figure 8), which suggested the association turned out to be invalid in the allele model and more studies are required for the recessive and heterozygous model. Regarding CILP (1184T>C) polymorphism and RIDD, the cumulative z-curve reached the TSA line in the allele and recessive model (Figure 9). The evidence is adequate to confirm this significant association.

## Discussion

AS a multifactorial disease, IDD is thought to be mainly caused by overloading and senescence (Rannou et al., 2004; Zhang et al., 2009). In a broad sense, IDD contains multiple potential phenotypes such as SIDH, asymptomatic disc herniation, disc bulge, disc height or signal change, and Modic change. SIDH characterized by sciatica represents a more serious phenotype of IDD because over 70% of individuals with disc herniations are asymptomatic (Boden et al., 1990). Recently, mounting evidence highlights the function of genetic factors in the initiation and progression of IDD. TT genotype for IL-1α rs1800587 induced the overexpression of IL-1 (Dominici et al., 2002). The relationship between IL-1α rs1800587 polymorphism and IDD susceptibility has been investigated in numerous studies, but the results were inconsistent. Karppinen et al. and Chen et al. found that IL-1α polymorphism (rs1800587) was associated with the susceptibility of IDD in Finland and Chinese populations (Karppinen et al., 2009; Chen et al., 2018). However, the findings were not detected in Caucasian and Iranian populations (Paz Aparicio et al., 2011; Abdollahzade et al., 2018). CILP (1184T>C) polymorphism resulted in inhibition of the extracellular matrix proteins synthesis (Seki et al., 2005; Min et al., 2010). However, updated studies failed to detect the statistical significance in the Chinese and Finland populations (Virtanen et al., 2007). In addition, studies revealed the C allele as a protective factor in Finnish and Indian populations, which contrasts with studies in the Japanese population (Kelempisioti et al., 2011; Harshitha et al., 2018). Thus, updated research with new evidence was performed to obtain a constructive conclusion.

In our meta-analysis, significant associations were detected between IL-1α rs1800587 polymorphism and IDD and SIDH but more evidence is required to confirm the conclusion. The subgroup analysis confirmed that IL-1α rs1800587 polymorphism was correlated with the risk of both RIDD (Solovieva grade) and SIDH but not RIDD (Modic change). Thus, we assumed that the change of expression due to rs1800587 polymorphism might mainly participate in the degeneration of NP instead of the endplate. Moreover, the homozygous model had the highest risk score. Compared with RIDD, a stronger association was detected between IL-1α(+889C/T) and SIDH. The results highlighted the importance of IL-1α polymorphism in the occurrence and progression of IDD.

Moreover, the pooled ORs and 95% CIs and TSA result demonstrated that CILP (1184T>C) was a credible susceptibility variant for RIDD (Pfirrmann grade) instead of SIDH, indicating the genetic effect involved in the initial stage of NP degeneration. Consistent with the results of IL-1α(+889C/T), the highest risk score was detected in the homozygous model. Furthermore, CILP rs2073711 is associated with SIDH in an ethnicity-dependent manner, which was mainly due to the difference in allele frequencies for ethnicity. The C allele was found to be a protective factor for Caucasians but a risk factor for Asians. Additionally, the C allele is also a protective factor for knee arthritis in the European population (Valdes et al., 2004), which is consistent with this conclusion.

To our best knowledge, this research is the first to comprehensively assess the genetic effect of IL-1α(+889C/T) and CILP (1184T>C) in different phenotypes of IDD. The significant associations were evaluated using the FPRP test and TSA. All eligible data was extracted to provide more convincing evidence. Our findings provided a novel perspective on the relationship between genetic polymorphisms of IL-1α and CILP and IDD phenotype-dependent susceptibility and emphasized its role in IDD early screening and SIDH prevention. Population with high-risk genotypes should avoid longtime overloading and other risk factors leading to the development of IDD.

However, limitations should be claimed. First, despite a larger sample size in this study compared with previous studies, samples with other phenotypes of IDD are required to estimate the false-positive result. Second, this study did not estimate multiple confounding factors such as gender, occupation, and environmental factors, which might affect the effect estimates. Third, candidate gene studies have limitations in dealing with complex disorders and the results are often unrepeatable (Arango, 2017). A large-scale meta-analysis is a promising approach to solving the defect. Additionally, the potential value of polygenic risk score analysis for complex diseases has been highlighted (Arango, 2017). Even so, it is crucial to conduct individual studies because individual datasets are the basis of large-scale research and certain hypotheses may be better addressed in smaller studies, at least initially (Thompson et al., 2017).

## Conclusion

The current meta-analysis illustrated adequate evidence that CILP (1184T>C) polymorphism was significantly associated with the susceptibility of RIDD. However, the significant associations between IL-1α(+889C/T) and RIDD and SIDH were not noteworthy. Further research with larger sample size and various populations is required to verify the outcome.

## Data Availability

The original contributions presented in the study are included in the article/Supplementary Material, further inquiries can be directed to the corresponding author.
