# Supplementary material for: Association between CILP and IL-1α polymorphisms and phenotype-dependent intervertebral disc degeneration susceptibility: A meta-analysis
Source: Front Genet. 2022 Oct 6;13:1005393. doi: 10.3389/fgene.2022.1005393 (PMC9582649; doi:10.3389/fgene.2022.1005393)
Supplement: Supplementary file 1 [file Table1.DOCX]

| **Table 1 General characteristics and quality assessment of included studies** | | | | | | | | | | |
| --- | --- | --- | --- | --- | --- | --- | --- | --- | --- | --- |
| **Study** | **Year** | **Adequate definition of cases** | **Representativeness of cases** | **Selection of control subjects** | **Definition of control subjects** | **Control for important factor or additional factor** | **Exposure assessment** | **Same method of ascertainment for all subjects** | **Non-response rate** | **NOS** |
| **CILP(1184T>C)** |  |  |  |  |  |  |  |  |  |  |
| **Kelempisioti et al** | **2011** | **1** | **1** | **1** | **1** | **1** | **1** | **1** | **0** | **7** |
| **Seki et al** | **2005** | **1** | **1** | **1** | **1** | **1** | **1** | **1** | **0** | **7** |
| **Virtanen(a) et al** | **2007** | **1** | **1** | **1** | **1** | **1** | **1** | **1** | **0** | **7** |
| **Virtanen(b) et al** | **2007** | **1** | **1** | **1** | **1** | **1** | **1** | **1** | **0** | **7** |
| **Min et al** | **2009** | **1** | **1** | **1** | **1** | **1** | **1** | **1** | **0** | **7** |
| **Min et al** | **2010** | **1** | **1** | **1** | **1** | **1** | **1** | **1** | **0** | **7** |
| **Bhat et al** | **2018** | **1** | **1** | **1** | **1** | **1** | **0** | **1** | **0** | **6** |
| **IL-1α(+889C/T)** |  |  |  |  |  |  |  |  |  |  |
| **Solovieva(a) et al.** | **2004** | **1** | **1** | **1** | **1** | **1** | **1** | **1** | **0** | **7** |
| **Solovieva(b) et al.** | **2004** | **1** | **1** | **1** | **1** | **1** | **1** | **1** | **0** | **7** |
| **Karppinen et al.** | **2009** | **1** | **0** | **1** | **1** | **1** | **1** | **1** | **0** | **6** |
| **Aparicio et al.** | **2011** | **1** | **0** | **1** | **1** | **1** | **1** | **1** | **0** | **6** |
| **Serrano et al.** | **2014** | **1** | **1** | **1** | **1** | **1** | **1** | **1** | **0** | **7** |
| **Chen et al.** | **2018** | **1** | **1** | **1** | **1** | **0** | **1** | **1** | **0** | **6** |
| **Abdollahzade et al.** | **2018** | **1** | **1** | **1** | **1** | **1** | **1** | **1** | **0** | **7** |
| **a,b denote an independent study in the same article, respectively.** | | | | | | | | | | |
